# Monoclonal gammopathy of renal significance: An atypical presentation of Waldenström’s disease 

**DOI:** 10.5414/CNCS111200

**Published:** 2024-02-08

**Authors:** Pablo Rodríguez-Doyágüez, Motornaya-Morozova, Patricia Martínez-Miguel, Carolina Castillo-Torres, Óscar Toldos-González, Juan José Gil-Fernández

**Affiliations:** 1 Nephrology Department,; 2 Hematology Department,; 3 Pathology Department,University Hospital Príncipe de Asturias, Alcalá de Henares, and; 4 Pathology Department, University Hospital 12 de Octubre, Madrid, Spain

**Keywords:** Waldenström’s macroglobulinemia, cryoglobulinemia, glomerulonephritis, bendamustine

## Abstract

Waldenström’s disease is a rare lymphoproliferative syndrome in the bone marrow and sometimes in lymphoid organs which secretes high amounts of monoclonal immunoglobulin M into serum. It can remain indolent for years and rarely affects the kidney, with intraglomerular rather than intratubular damage being predominant, in contrast to multiple myeloma. Different studies identified AL amyloidosis as the most frequent renal lesion, followed by cryoglobulinemic glomerulonephritis. Signs and symptoms may be unspecific, as well as renal manifestations, so collaboration between nephrologists, hematologists, and pathologists is crucial to establish the role of paraprotein in the development of renal damage. We present an atypical case of Waldenström’s disease that had a minimal monoclonal peak and clinically debuted with nephritic and nephrotic syndromes. The diagnosis was cryoglobulinemic glomerulonephritis. Currently, there are numerous treatment options, without enough evidence yet to establish a standardised treatment.

## Introduction 

Waldenström’s disease (WD) is a lymphoproliferative syndrome characterized as a lymphoplasmacytic lymphoma in the bone marrow (BM) and sometimes in lymphoid organs, in which tumor B cells can synthesize and secrete high amounts of monoclonal immunoglobulin M (IgM) into serum. It is a rare entity accounting for only 2% of all non-Hodgkin’s lymphomas [1, 2, 3, 4]. Renal involvement in this disease, although described, is rare. We present a case of WD with a minimal monoclonal peak, whose initial clinical manifestation was nephritic and nephrotic syndromes, and the diagnosis resulted in cryoglobulinemic glomerulonephritis (CGN). The deposition of monoclonal IgM in the glomerular capillaries can lead to atypical cases such as this one. Targeted treatment of WD (indicated by renal involvement) with the combination of bendamustine and rituximab achieved a satisfactory clinical and biological response. 

## Case report 

We describe the case of a 48-year-old woman with a history of iron deficiency anemia, referred to the nephrology department for hypertension and edema. She presented signs of nephrotic syndrome (proteinuria of 6.5 g/24h with hypoalbuminemia and hyperlipidemia) and nephritic syndrome (hypertension, microhematuria, and mild deterioration of renal function with creatinine of 1.59 mg/dL and eGFR according to CKD-EPI of 38 mL/min/1.73m^2^). She had no purpuric skin lesions. The immunological study showed a monoclonal IgM-λ peak in serum (0.58 g/dL) and in urine, elevated IgM in serum (1,110 mg/dL) and low C3c levels (55 mg/dL, normal range 75 – 144 mg/dL) with C4 in the lower limit (10.8 mg/dL, normal range 10 – 40 mg/dL); autoimmunity studies, serology for hepatitis B and C, and HIV viruses and serum cryoglobulins were negative. 

**Figure 1.**
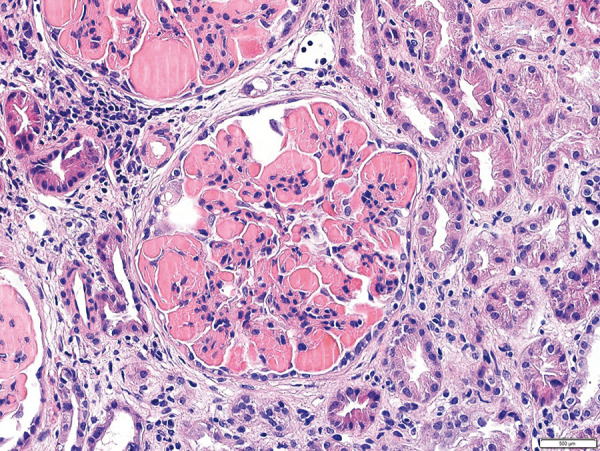
Optical microscopy: glomeruli with nodular pattern, with massive eosinophilic deposits in capillaries. The areas without deposits showed increased cellularity and mesangial matrix, with polymorphonuclear leukocytes and monocytes, and focal images of double contours.

**Figure 2.**
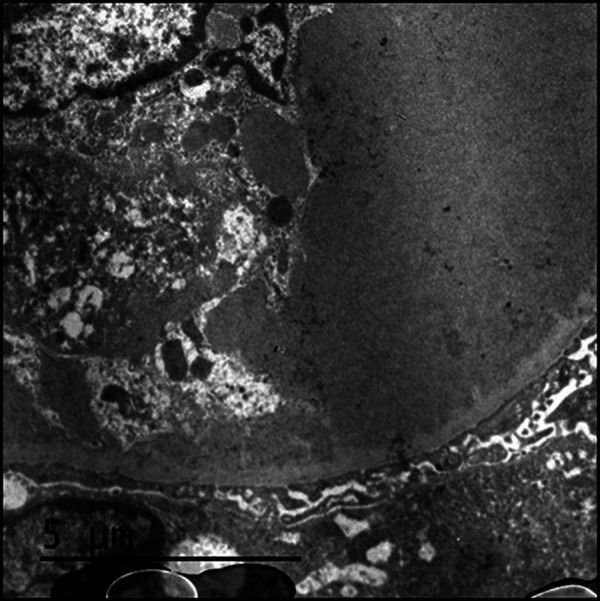
Electron microscopy: fibrillar material with microtubular foci in the intracapillary and subendothelial region, compatible with cryoglobulin deposits.

Renal biopsy showed 15 enlarged glomeruli with nodular pattern, with massive eosinophilic deposits in capillaries, positive for PAS technique and negative for Masson and Jones staining. The areas without deposits showed increased cellularity and mesangial matrix, with polymorphonuclear leukocytes and monocytes, and focal images of double contours (Figure 1). Immunofluorescence (IF) showed fine and coarse granular deposits in capillaries and mesangium, with IgM (+++), IgA (+), C3 (++), C1q (+), κ (+), and λ (+++). Electron microscopy (EM) showed fibrillar material with microtubular foci in the intracapillary and subendothelial region, compatible with cryoglobulin deposits (Figure 2). The anatomopathological diagnosis was CGN. 

Given these results, treatment with steroids 1 mg/kg/day was started to stop the immunological activity at the renal level, and the patient was referred to the hematology department for further evaluation. A body scan showed moderate pericardial effusion, no organomegaly, and no adenopathic involvement suggesting a lymphoproliferative process. 

The BM aspirate showed a scanty lump and normal cellularity, with heterogeneous megakaryocytes, normal in number. The granulocytic series was correctly represented in all stages, without maturation arrest or significant dysplasia. The erythroid series was slightly decreased, without significant morphological alterations, with slightly increased lymphocytes (16%) without atypia, and without alterations in plasma cells or in the phagocytic mononuclear system. The BM biopsy showed a slight increase of lymphoplasmacytoid cells of interstitial distribution, in a percentage of less than 10%, with a slight restriction of λ light chains by immunohistochemistry. In cell culture, all metaphases analyzed by GTG bands showed 46 chromosomes with normal female formula XX and no structural alterations. In the molecular biology of BM, lymphoid B clonality was detected in the CDR1 region of the *IgH* gene, and the pLeu265Pro mutation of the *MYD88* gene was also detected, establishing the diagnosis of WD. 

With the diagnosis of WD and given the aggressiveness of the disease manifestations at the renal level, it was decided to revaccinate against SARS-CoV-2 (the third dose of the vaccine due to the absence of humoral immune response after the two previous doses) and to initiate treatment with 2 cycles of bendamustine and later 4 cycles of bendamustine-rituximab. The evolution was remarkably satisfactory, with normalization of renal function and disappearance of nephrotic and nephritic syndrome (Table 1). 

## Discussion 

We present an unusual case of WD with renal involvement with no other clinical manifestations and a monoclonal IgM deposit, consistent with a CGN without confirmation of positive cryoglobulins in blood. Clinical manifestations in WD may take years to appear. The marked increase in monoclonal IgM levels leads to increased blood viscosity with neurological, cutaneous, respiratory, pulmonary, or gastrointestinal manifestations [4]. Most patients with WD have symptoms and signs related to tumoral infiltration (cytopenia and hepatosplenomegaly), monoclonal protein accumulation in circulation (cryoglobulinemia and serum hyperviscosity syndrome), monoclonal protein accumulation in the tissues (amyloidosis), and/or autoantibody production (neuropathy and hemolytic anemia). The most common presentation is fatigue due to normocytic anemia. Less than 8% of patients with WD present with renal involvement. However, various glomerular and tubular lesions have been described in patients with WD, with glomerular involvement predominating over tubular damage, in contrast to multiple myeloma [4, 5]. Kidney complications in multiple myeloma are well described, but the spectrum of renal pathology in WD has received less attention, probably due to the infrequency of this disease. 

A study performed by the Mayo Clinic evaluated 57 patients with WD and other IgM-secreting lymphoproliferative disorders undergoing renal biopsies [6]. It identified amyloidosis glomerulopathy as the most frequent renal lesion (33%, n = 19), with Ig light chain amyloidosis being the most numerous subtype. Different types of non-amyloid paraprotein glomerulopathy were found in 20 biopsies, and the most frequent was cryoglobulinemic glomerulonephritis (20%, n = 12). The third most frequent finding in the series was lymphoma infiltration. Of all the results, pathology related only to monoclonal gammopathy was observed in 47 of the cases (82%). Other older series obtained similar percentages, also with a varied histopathological spectrum but with amyloidosis, cryoglobulinemic glomerulonephritis, and lymphoma infiltration as the most frequent findings [7, 8]. 

Cryoglobulins are immunoglobulins that precipitate at temperatures below 37 °C and occlude the capillary lumen [4, 9, 10]. They are classified according to clonality into type I, if they are monoclonal proteins; type II, if there is a monoclonal and polyclonal peak; and type III, if the proteins are polyclonal. Type I is usually associated with lymphoproliferative disorders, whereas types II and III are associated with infections (such as active or hidden hepatitis B virus [9] or hepatitis C virus) or autoimmune diseases [4, 5, 7, 9, 10]. Cryoglobulinemic glomerulonephritis in WD less frequently presents as nephrotic syndrome compared to amyloid glomerulopathy, and more commonly with hypertension and microhematuria [6]. Renal biopsy shows a pattern of membranoproliferative glomerulonephritis along with PAS-positive intracapillary pseudothrombi, which are these cryoglobulins or intracapillary aggregates of monoclonal IgM. Some of these patients are diagnosed with type I or II – III cryoglobulinemia [4, 6]. IF shows dominant monoclonal IgM staining, with specificity for κ or λ light chains, involving the mesangium, the walls of glomerular capillaries or they can be seen as intraluminal pseudothrombi. IF is useful to differentiate hyaline thrombi due to deposits (pseudothrombi) from actual thrombi, because cryoglobulinemic pseudothrombi show negative fibrin staining and positive Ig staining, while the opposite result supports a real thrombus [5]. EM typically shows subendothelial deposits with or without substructural organization, which may have fibrillar and microtubular features [5, 8, 9, 11]. The rest of the renal manifestations of WD may also present intracapillary thrombi, although they usually lack a defined structure [9]. 

The diagnosis of our patient raised doubts, starting from a monoclonal gammopathy of renal significance and without a diagnosis of WD until the cytogenetic result was known. Although the anatomopathologic result was cryoglobulinemic glomerulonephritis: 1) the patient had no signs or symptoms of cryoglobulinemia like skin manifestations (ulcers, livedo reticularis, purpuric rash, etc.), Raynaud’s phenomenon or acrocyanosis, arthralgias or peripheral neuropathy 2) and serum cryoglobulins were negative; so clinically we cannot consider that she had an associated cryoglobulinemia [6, 10]. 

Treatment in WD is aimed at reducing tumor burden and eliminating circulating IgM [4] and is recommended if there are symptoms related to hyperviscosity, venous thrombosis, or extension of the disease with organ involvement. Otherwise, a watchful waiting approach is preferred [5]. There is currently no standard treatment approach [5, 6, 12]. Plasmapheresis is often used in patients with symptoms of hyperviscosity or cryoglobulinemia, until systemic therapy successfully lowers the tumor mass and reduces the IgM protein concentration in the serum [13]. The systemic therapy includes different combinations of corticosteroids, alkylating agents, rituximab, proteasome inhibitors, or autotransplantation of hematopoietic progenitors. Rituximab-based immunochemotherapy regimens were the mainstay of treatment for first-line and relapsed/refractory WD, but promising new therapies such as Bruton’s tyrosine kinase inhibitor (BTKi) or bendamustine have been developed with favorable safety and efficacy profiles [5, 6, 10, 13, 14, 15]. 

Bendamustine-rituximab has shown excellent hematological response rates with longer progression-free survival compared with other options like R-CHOP or cyclophosphamide-dexamethasone-rituximab [13]. Added to its low rates of non-hematologic adverse events, limited time of therapy (usually less than 6 months) and no risk of IgM flare (transient rise in the level of IgM when rituximab is initiated, producing an increase of serum viscosity), bendamustine-rituximab has become one of the Mayo Clinic’s first-line induction treatments for newly diagnosed and relapsed/refractory WD, along with BTKi (especially zanubrutinib) [13]. Due to the fact that bendamustine-rituximab could be used in a cyclic manner (allowing us to interrupt it once the response at the renal level is achieved), we decided to start with it. Although BTKi was another excellent option with similar overall survival and progression-free survival rates specially in the MYD88 genotype (in fact, Mayo Clinic’s algorithm also recommends it as a first-line or relapse option) [13], we decided to leave zanubrutinib for an eventual relapse given the initial picture of nephritic syndrome with hypertension and the cardiovascular risks that have been associated with this group of drugs (like hypertension and atrial fibrillation) [13, 14]. A recent systematic review and meta-analysis suggests that bendamustine-rituximab yields higher response rates compared to other first-line treatment options [15]. 

The follow-up of renal function after initiation of treatment for WD was biweekly. In the case of our patient (Table 1), renal parameters improved as she responded to the hematologic treatment, and follow-up could be spaced from the second month onwards. The improvement of the nephrotic and nephritic syndrome with normalization of the blood pressure was quite fast. The evolution of the anemia was also satisfactory, with little need for erythropoietin. Serum IgM did not change significantly. We believe that the follow-up should be individualized in relation to the degree of renal involvement at diagnosis and the possible risk factors of the patient such as age, comorbidity, or the presence of previous chronic kidney disease. There are few data available in the literature on renal prognosis in WD and the time at which we expect renal parameters to improve, finding a direct relationship between hematologic prognosis (complete or very good response) and renal prognosis, as well as longer survival in patients with improved or stable renal function [5]. Patients with amyloid-related glomerulopathy had the highest rate of progression to end-stage renal disease [6]. 

This case showed the importance of close collaboration between nephrologists, hematologists-oncologists, and pathologists to establish the role of paraprotein in the development of nephrotic/nephritic syndrome and renal function impairment and to be able to design an early treatment targeting the lymphoid or plasmacellular neoplasm-originating monoclonal gammopathy of renal significance. 

## Funding 

None. 

## Conflict of interest 

The authors declare no conflict of interest. 

**Table 1.** Evolution just after initiation of treatment, with normalization of renal function and disappearance of nephrotic and nephritic syndrome.

	**11/08/2021**	**12/07/2021**	**01/05/2022**	**01/26/2022**	**03/10/2022**	**06/15/2022**
Hemoglobin (g/dL)	10.5	10.3	10.4			12.5
Creatinine (g/dL)	1.59	1.55	1.21	1.26	1.28	0.86
Estimated glomerular filtration rate (CKD-EPI, mL/min/1.73 m^2^)	38.13	39.33	53.05	50.03	49.56	80.16
Proteinuria (g/24h)	6,828	2,993	3,742	2,156	248	254
Urine red blood cells (red blood cells per high power field)	30 – 50	30 – 50	30 – 50	20 – 30	1 – 5	1 – 3
IgM peak (g/dL)	0.58	0.49	0.32	0.35	0.41	0.4
κ free chains (mg/L)	22.5	20.97	20.45	25.26	26.74	18.17
λ free chains (mg/L))	16.92	18.45	16.28	18.2	18.11	13.28
κ/λ ratio	1.33	1.13	1.26	1.39	1.48	1.37
